# Association between media exposure and family planning in Myanmar and Philippines: evidence from nationally representative survey data

**DOI:** 10.1186/s40834-021-00154-9

**Published:** 2021-04-01

**Authors:** Pranta Das, Nandeeta Samad, Hasan Al Banna, Temitayo Eniola Sodunke, John Elvis Hagan, Bright Opoku Ahinkorah, Abdul-Aziz Seidu

**Affiliations:** 1 Department of Statistics, University of Dhaka, Dhaka, Bangladesh; 2 Department of Public Health, North South University, Dhaka, Bangladesh; 3 Institute of Social Welfare and Research, University of Dhaka, Dhaka, Bangladesh; 4 Bachelor of Science in Anatomy, University of Ilorin, Ilorin, Nigeria; 5 Department of Health, Physical Education, and Recreation, University of Cape Coast, Cape Coast, Ghana; 6 Neurocognition and Action-Biomechanics-Research Group, Faculty of Psychology and Sport Sciences, Bielefeld University, Bielefeld, Germany; 7 School of Public Health, Faculty of Health, University of Technology Sydney, Ultimo, Australia; 8 Department of Population and Health, University of Cape Coast, Cape Coast, Ghana; 9 College of Public Health, Medical and Veterinary Sciences, James Cook University, Townsville, Queensland Australia

**Keywords:** DHS, Family planning, Media exposure, Myanmar, Philippines, Women’s health

## Abstract

**Background:**

Although women in South Asia and South-east Asia have developed their knowledge regarding modern contraceptive and other family planning techniques, limited information exists on the influence of mass media exposure on the utilization of contraceptives and family planning. The current study examined the association between media exposure and family planning in Myanmar and Philippines.

**Methods:**

The study analyzed data from the 2017 Philippines National Demographic and Health Survey (NDHS) and 2015–16 Myanmar Demographic and Health Survey (MDHS). Three family planning indicators were considered in this study (i.e., contraceptive use, demand satisfied regarding family planning and unmet need for family planning). A binary logistic regression model was fitted to see the effect of media exposure on each family planning indicator in the presence of covariates such as age group, residence, education level, partner education level, socio-economic status, number of living children, age at first marriage, and working status.

**Results:**

The prevalence of contraception use was 57.2% in the Philippines and 55.7% in Myanmar. The prevalence of demand satisfied regarding family planning was 70.5 and 67.1% in the Philippines and Myanmar respectively. Unmet need regarding family planning was 16.6% and 19.9% in the Philippines and Myanmar respectively. After adjusting for the covariates, the results showed that women who were exposed to media were more likely to use contraception in Philippines (aOR = 2.24, 95% CI = 1.42–3.54) and Myanmar (aOR 1.39, 95% CI = 1.15–1.67). Media exposure also had a significant positive effect on demand satisfaction regarding family planning in the Philippines (aOR = 2.19, 95% CI = 1.42–3.37) and Myanmar (aOR = 1.34, 95% CI = 1.09–1.64). However, there was no significant association between media exposure and unmet need in both countries.

**Conclusions:**

The study established a strong association between mass media exposure and the use and demand satisfaction for family planning among married and cohabiting women in Philippines and Myanmar. Using mass media exposure (e.g., local radio, television- electronic; newspapers) to increase both access and usage of contraceptives as well as other family planning methods in these countries could be pivotal towards the attainment of United Nations Sustainable Development Goal 3 (SDG 3) of improving maternal health.

## Background

Globally, it has been observed that family planning issues are highly influenced by the scientific use of mass media, especially television, radio, newspaper, and internet [1]. Similarly, the last three decades have shown that indicators of family planning such as contraceptive use, unmet need for family planning, and demand satisfied regarding family planning have significant association with media exposure [1, 2]. Furthermore, the world has noticed an increased trend regarding these indicators of family planning. For example, worldwide data in 2017 indicates that the rate of contraceptive use among married or in-union women of reproductive age rose to 63% from 35% in 1970. Likewise, an increased trend (78% from 75% in 2000) has also been observed concerning the demand for family planning satisfied by modern methods among married or in-union women. However, 12% of women have an unmet need for family planning, which has declined from 22% in 1970 [3, 4]. A study suggests that the SMS-based communication coverage regarding family planning is higher in Africa than Asia [5]. However, the percentage in terms of contraceptive use in Central and West Africa is very low (25%) and in Asia, the rate is 66.4%, which is considered low compared to Thailand, Vietnam, and Singapore [6–8].

It is evident that women in South Asia and South-East Asia have developed their knowledge regarding modern contraceptive techniques through the proper utilization of mass media [9, 10]. For instance, in India, Pakistan, and Philippines, women who own TV at their homes are more likely to get access in the coverage of contraceptives than women who do not have TV [11–13]. Similarly, exposure of mass media in Myanmar is associated with the age of women at first marriage [14, 15]. A study conducted from 47 DHS data of sub-Saharan Africa indicates that overall, 44.3% family planning related information is available from mass media [16]. Furthermore, in Nigeria, in terms of getting family planning related messages, people with higher socio-economic status have more opportunity of getting access to mass media, especially television and radio, than people with lower socio-economic status [17, 18]. In Indonesia, the exposure of television has increased than radio and print media regarding family planning [19]. Also, in Ethiopia, women’s access to mass media, especially radio, television and newspaper has a significant impact on unmet need for child spacing and limiting births. For instance, data from the year 2005 in Ethiopia proved that women with no access to mass media had higher rate (38.1%) of unmet need for family planning than women with access to mass media (35.8%) [20]. In addition, some covariates like education, partner education, residence, and occupation also had significant impact on family planning. Specifically, urban women were more likely to utilize knowledge about contraceptive use from mass media than rural women. Similarly, educated women were more likely to obtain health related messages through mass media at their homes than healthcare centers [21].

Despite the numerous studies on family planning issues worldwide, these studies have paid more attention to the impact of basic socio-demographic status such as education, economic condition, and decision-making power on family planning rather than recognizing the importance of mass media for awareness building regarding family planning [19, 22–29]. Hence, the literature in Asia regarding media exposure and family planning is sparse. Although there is limited scholarly information on this issue, the influence of media exposure on family planning remains unclear [30, 31]. By utilizing the current DHS data, it is possible to present a valid outcome regarding this prevailing issue. Myanmar and Philippines were targeted for this study because in Myanmar, the contraceptive prevalence rate (CPR) was lower (41.0%) than other Southeast Asian countries (average rate of this region was 62.2% in 2007) [32]. Although the CPR increased at 52.2% in 2016, the rate was lower than a projected  target of 60% by 2020 [33]. Rapid population growth, short life expectancy, low level of education, and poor healthcare system are the major loopholes in the process of achieving the goal of population control and ensure a sound maternal and child health by preventing unwanted pregnancies and encouraging birth spacing [14, 34]. Moreover, the rate of unmet need for family planning in Myanmar was very high (19%) than their nearest country, Thailand (3%) [34, 35]. Likewise, the population growth rate was highest in Philippines among the South-East Asia, along with the third highest total fertility rate (TFR) in the region. UN 2015 data indicated that the rate of unmet need of FP was 17.8%, which was higher than other Asian countries [36]. Furthermore, the overall contraceptive prevalence was only 40% among married women in 2017 in Philippines, where the rate of modern contraceptive prevalence increased by only 2% between 2013 and 2017 [37]. Therefore, evidence suggests these countries have challenges to increase the prevalence of contraceptive use and reduce the TFR [38]. This study, therefore, examines the association between media exposure and family planning in both Myanmar and Philippines. By making this assessment between these two countries, researchers and policy makers could get updated view on reproductive health issues for making proper guidelines and efficient programs.

## Materials and methods

### Data source and study settings

The study analyzed the 2017 Philippines National Demographic and Health Survey (NDHS) dataset, which took place from August 14 to October 27, 2017, and 2015–16 Myanmar Demographic and Health Survey (MDHS) dataset, which took place from December 7, 2015 to July 7, 2016. These two datasets were selected because they were the most recent dataset available for both countries.

### Study design

The 2017 Philippines National Demographic and Health Survey (NDHS), implemented by the Philippine Statistics Authority (PSA), was based on two-stage stratified sample design. It used the Master Sample Frame (MSF) which was designed as well as compiled by the PSA. In the first stage, 1250 primary sampling units (PSUs), distributed by province, were systematically selected. Systematic random sampling from each sampled PSU 20 or 26 housing units were selected at the second stage. Then, from each housing unit, women aged 15–49 were interviewed. To prevent bias replacement, changes in the pre-specified housing units were not allowed. After the data collection, information of 25,074 women were obtained.

The 2015–16 Myanmar Demographic and Health Survey (MDHS) was implemented by the Ministry of Health and Sports (MoHS). This survey followed a two-stage stratified sample design. From the master sample, 442 clusters (123 urban and 319 rural) were selected at the first. At the second stage, by using equal probability systematic sampling from each selected clusters (i.e., a total of 13,260 households), 30 households were selected. Then, from each household, women aged 15–49 were interviewed. After the data collection, data of total 12,885 women were obtained.

The data of women who were married or currently in union and were usual residents and not pregnant, and with education level, including partner’s education level were filtered out from the raw dataset of both countries. This filtered dataset was subsequently analyzed.

### Dependent variables

The indicators of family planning selected were contraception use, unmet need for family planning and demand satisfied regarding family planning. The variable contraception use was coded as “Yes” for a woman who was currently using any kind of contraception method, otherwise coded as “No” and the variable unmet need for family planning was coded as “Yes” for a woman who had an unmet need for spacing or unmet need for limiting otherwise “No”. For a woman who had met the need for spacing or met the need for limiting, the indicator demand satisfied regarding family planning was coded as “Yes” otherwise “No”.

### Independent variable

The variable media exposure was recoded as ‘Yes’ for a woman who was exposed to either newspaper, radio, television or the internet otherwise “No” for both the countries (Philippines, Myanmar).

A number of covariates were selected for this study based on previous literature [36–46]. These are age group (15–24, 25–29, 30–34, 35–39, 40–49), residence (urban, rural), education level (no education, primary, secondary, higher), partner education level (no education, primary, secondary, higher), socio-economic status (poorest, poorer, middle, richer, richest), number of living children (no children, 1–3, more than 3), age at first marriage (below 20, 20–25, more than 25), and working status (employed, unemployed).

### Statistical analysis

After the required filtration of the dataset, chi-square was performed between the confounders and the family planning indicators. The binary logistic regression model was fitted to see the association between media exposure with each family planning indicators in the presence of covariates age group, residence, educational level, partner’s educational level, socio-economic status, number of living children, age at first marriage, and working status. In each model, the covariates were kept regardless of their *p*-value. Each model was assessed by calculating the area under the ROC curve and were  found satisfactory. The analysis was done after considering the sample weight and the complex survey design. All the *p*-values less than 0.05 were considered significant. The analysis was conducted using software R version 3.6.0.

## Results

### Sample characteristics

The weighted sample size for Philippines and Myanmar was 4497 and 7047 respectively. For both countries, most of the women were in the age group 40–49 years (see Table 1). For Philippines, 60.2% of the women were from urban areas whereas 26.3% of the women in Myanmar were from urban areas. The sample from Philippines comprised 46.4% of women with a secondary level of education and 41.1% women with partner having secondary level of education whereas the sample of Myanmar comprised 29% women with a secondary level of education and 38.2% women with partner having secondary level of education. The socio-economic status of the women of Philippines was mostly in the  richest quintile whereas women from Myanmar have socio-economic status mostly in the middle and poorer quintile. Most of the women of Philippines had age at first marriage within 20–25 while most of the women of Myanmar had age at first marriage below 20. For both countries, most of the women had 1–3 living children. Also, majority of the women from Philippines were employed whereas most of the women of Myanmar were unemployed.

**Table 1:** General characteristics of the respondents

Variable	Philippines (4497)		Myanmar (7047)	
	N	Percent	N	Percent
Age group				
• 15–24	543	12.1	860	12.2
• 25–29	750	16.7	1057	15
• 30–34	765	17	1353	19.2
• 35–39	851	18.9	1395	19.8
• 40–49	1588	35.3	2382	33.8
Residence				
• Urban	2706	60.2	1854	26.3
• Rural	1791	39.8	5193	73.7
Education level				
• No education	51	1.1	1097	15.6
• Primary	693	15.4	3348	47.4
• Secondary	2087	46.4	2041	29
• Higher	1666	37.1	561	8
Partner education level				
• No education	59	1.3	1063	15.1
• Primary	934	20.8	2850	40.4
• Secondary	1849	41.1	2689	38.2
• Higher	1655	36.8	445	6.3
Socio-economic status				
• Poorest	780	17.3	1423	20.2
• Poorer	775	17.2	1446	20.5
• Middle	880	19.6	1442	20.5
• Richer	1022	22.7	1373	19.5
• Richest	1040	23.2	1363	19.3
Number of living children				
• No children	341	7.6	496	9.9
• 1–3	3025	67.3	5022	71.3
• More than 3	1131	25.2	1328	18.8
Age at first marriage				
• Below 20	1763	39.2	3322	47.2
• 20–25	1909	42.5	2674	37.9
• Above 25	825	18.3	1051	14.9
Working status				
• Employed	2300	51.1	2458	34.9
• Unemployed	2197	48.9	4588	65.1

**Fig. 1:**
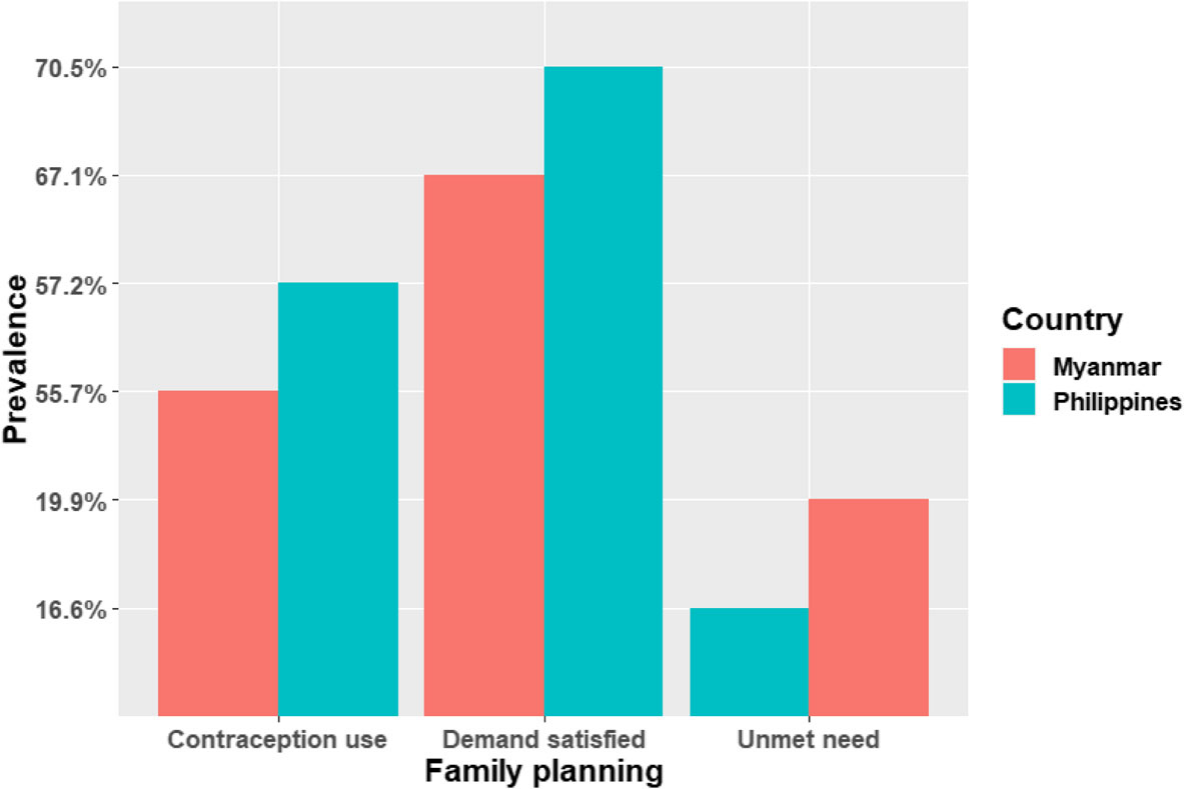
Prevalence of family planning indicators

The family planning indicator contraception use prevalence was 57.2% in the Philippines and 55.7% in Myanmar (see Fig. 1). The prevalence of demand satisfied regarding family planning was 70.5 and 67.1% in the Philippines and Myanmar respectively. Unmet need regarding family planning was 16.6% and 19.9% in the Philippines and Myanmar respectively.

### Chi-square test between the confounding variables and family planning indicators

The results of the chi-square test and the distribution of the family planning indicators across the confounding variables are presented in the Tables 2, 3, 4.

**Table 2:** Chi-square test of the association between contraception use and confounders

Variable	Philippines			Myanmar		
	Contraception use		*p*-value	Contraception use		*p*-value
	Yes	No		Yes	No	
Age group						< 0.01
• 15–24	339	203		580	280	
• 25–29	454	297		710	330	
• 30–34	486	280	< 0.01	834	522	
• 35–39	530	320		923	469	
• 40–49	765	824		880	1498	
Residence						
• Urban	1564	1142	0.529	1170	684	< 0.01
• Rural	1009	782		2757	2433	
Education level						< 0.01
• No education	10	41		443	633	
• Primary	355	337	< 0.01	1820	1527	
• Secondary	1313	774		1298	743	
• Higher	895	772		366	194	
Partner education level						< 0.01
• No education	10	41		429	634	
• Primary	355	337	< 0.01	1595	1255	
• Secondary	1313	774		1621	1068	
• Higher	895	772		283	162	
Socio-economic status						< 0.01
• Poorest	398	381		732	467	
• Poorer	494	281		774	412	
• Middle	534	346	< 0.01	765	391	
• Richer	610	412		802	339	
• Richest	537	504		854	314	
Number of living children						< 0.01
• No children	39	302		266	430	
• 1–3	1870	1154	< 0.01	3088	1934	
• More than 3	663	468		573	755	
Age at first marriage						< 0.01
• Below 20	1064	698		1872	1450	
• 20–25	1134	775	< 0.01	1552	1122	
• Above 25	374	451		503	548	
Working status						0.66
• Employed	1241	956	0.57	1381	1077	
• Unemployed	1332	968		2546	2042

**Table 3:** Chi-square test of the association between demand satisfied and confounders

Variable	Philippines			Myanmar		
	Demand satisfied		*p*-value	Demand satisfied		*p*-value
	Yes	No		Yes	No	
Age group						< 0.01
• 15–24	339	190		580	268	
• 25–29	454	240		710	313	
• 30–34	486	188	0.04	834	414	
• 35–39	530	172		923	334	
• 40–49	765	287		880	594	
Residence						< 0.01
• Urban	1564	601	0.07	1170	403	
• Rural	1009	476		2757	1520	
Education level						< 0.01
• No education	10	13		443	375	
• Primary	355	200	< 0.01	1820	895	
• Secondary	1313	438		1298	515	
• Higher	895	426		366	138	
Partner education level						< 0.01
• No education	23	22		429	393	
• Primary	494	260	< 0.01	1593	748	
• Secondary	1138	387		1621	677	
• Higher	918	408		283	106	
Socio-economic status						< 0.01
• Poorest	398	238		732	467	
• Poorer	494	174		774	412	
• Middle	534	203	< 0.01	765	391	
• Richer	610	199		802	339	
• Richest	537	263		854	314	
Number of living children						< 0.01
• No children	39	165		266	282	
• 1–3	1870	663	< 0.01	3088	1236	
• More than 3	663	249		573	405	
Age at first marriage						< 0.01
• Below 20	1064	431		1872	914	
• 20–25	1134	415	< 0.01	1552	676	
• Above 25	374	230		503	333	
Working status						0.37
• Employed	1241	572	0.06	1381	704	
• Unemployed	1332	505		2546	1219

**Table 4:** Chi-square test of the association between unmet need and confounders

Variable	Philippines			Myanmar		
	Unmet need		*p*-value	Unmet need		*p*-value
	Yes	No		Yes	No	
Age group						< 0.01
• 15–24	89	439		125	723	
• 25–29	100	594		156	867	
• 30–34	97	577	< 0.01	202	1046	
• 35–39	87	615		184	1073	
• 40–49	233	818		498	976	
Residence						< 0.01
• Urban	337	1829	0.20	246	1326	
• Rural	270	1214		919	3359	
Education level						< 0.01
• No education	7	16		261	557	
• Primary	128	427	0.01	570	2146	
• Secondary	261	1490		283	1529	
• Higher	211	1110		51	453	
Partner education level						< 0.01
• No education	13	31		263	558	
• Primary	151	603	0.07	484	1858	
• Secondary	227	1298		371	1926	
• Higher	216	1110		47	342	
Socio-economic status						< 0.01
• Poorest	134	501		292	907	
• Poorer	107	560		247	939	
• Middle	122	615	0.08	232	924	
• Richer	104	705		218	923	
• Richest	139	661		176	992	
Number of living children						< 0.01
• No children	15	189		59	489	
• 1–3	385	2148	< 0.01	778	3546	
• More than 3	207	706		328	650	
Age at first marriage						0.001
• Below 20	278	1217		616	2170	
• 20–25	231	1318	0.32	417	1811	
• Above 25	97	508		133	703	
Working status						0.50
• Employed	324	1489	0.18	404	1681	
• Unemployed	283	1554		762	3003	

### Binary logistic regression models between family planning indicators and media exposure

After adjusting for the covariates, the results showed that media exposure has a significant effect on contraception use for both Philippines and Myanmar (see Table 5). In Philippines, media exposed women are significantly 1.27 times more likely to use any contraception method than women who are not media exposed. Similarly, in Myanmar, media exposed women are significantly 39% more likely to use any contraception method than women who are not media exposed.

**Table 5:** Association between media exposure and contraceptive use after adjusting for the covariates

Variable	Philippines				Myanmar			
	aOR	95% CI	*P*-value	ROC curve	aOR	95% CI	*P*-value	ROC curve
Media Exposure								
• Yes	2.27	1.45 -3.56	< 0.001*	68.2%	1.39	1.15–1.67	0.001*	69.7%
• No	Ref.				Ref.			

Note: Reference category of contraception use is ‘No’

*CI* Confidence interval, *aOR* Adjusted odds ratio

*significant

Media exposure also has a significant effect on family planning indicator demand satisfied in the presence of all the covariates for both countries (see Table 6). In Philippines media exposed women are significantly 1.15 times more likely to have demand satisfied regarding family planning than the women who are not media exposed. Similarly, in Myanmar media exposed women are 34% more likely to have demand satisfied regarding family planning than the women who are not media exposed.

**Table 6:** Association between media exposure and demand satisfied regarding family planning after adjusting for covariates

Variable	Philippines				Myanmar			
	aOR	95% CI	*P*-value	ROC curve	aOR	95% CI	*P*-value	ROC curve
Media Exposure								
• Yes	2.15	1.41 -3.27	< 0.001*	65.5%	1.34	1.09 –1.64	0.006*	64.4%
• No	Ref.				Ref.			

Note: Reference category of demand satisfied is ‘No’

*CI* Confidence interval, *aOR* Adjusted odds ratio

*significant

But in the presence of all the covariates, the result shows that media exposure has no significant effect on family planning indicator unmet need for both countries (see Table 7).

**Table 7:** Association between media exposure and unmet need for family planning after adjusting for the covariates

Variable	Philippines				Myanmar			
	aOR	95% CI	*P*-value	ROC curve	aOR	95% CI	*P*-value	ROC curve
Media Exposure								
• Yes	0.79	0.55–1.15	0.23^ns^	60.6%	0.87	0.72 –1.06	0.18^ns^	65.4%
• No	Ref.				Ref.			

Note: Reference category of unmet need is ‘No’

*CI* Confidence interval, *aOR* Adjusted odds ratio, *ns* not significant

## Discussion

We examined the association between mass media exposure and family planning and compared the assessment in both countries so that researchers and policy makers could get an utter view for making proper guidelines and efficient programs. Using data from the Demographic and Health Survey in Philippines and Myanmar, the regression analysis was fitted to show the effect of mass media exposure on the various family planning indicators. After adjusting for covariates, the study revealed that mass media exposure has a significant effect on contraception use in both countries. In the Philippines, the results indicate that women who had media exposure were approximately 2 times more likely to use any contraceptive methods as opposed to their counterparts who were not exposed to mass media. Similarly, in Myanmar, mass media exposed women were 39% more likely to use any contraceptive method than women who were not exposed to mass media. This finding supports other studies that have documented the influence of mass media communication interventions on contraceptive use [2–9]. Additionally, studies [10–12] have claimed that mass media remains a vital source of information and has the capacity to raise awareness, increase knowledge level, and influence attitudes towards family planning. This same evidence aligns with another work [13] in Kenya that revealed that access to media messages influences the use of contraceptives, intention to use contraceptives, and even desire for future births.

Other studies have demonstrated that mass media impacts women empowerment, including their ability to take household decisions on contraception [47–51]. For example, sexual partners who access diverse media platforms (e.g., television, radio, newspapers), communicate more about safer sexual practices and subsequently use contraceptives (e.g., condoms), and/or other family planning methods. Thus, exposure to media could improve knowledge and attitudes through behaviour modification associated with consistent use of some contraceptives and other family planning methods. Some behaviours and communication change models emphasize that knowledge is one key prerequisite for a positive behavioural change [14–17, 52].

Another important finding in our study is the effect of mass media exposure on demand satisfied regarding family planning. Surprisingly, there is little published data on this aspect and its association with mass media. The current study reveals that mass media exposure also has a significant effect on satisfaction of demands in the presence of all the covariates for both countries. For both countries, geographical disparities or heterogeneity (e.g., population density, ageing pattern) in attitudes and norms, differentials in employment, and other socio-economic parameters (e.g., household wealth index) might mirror the accessibility of media platforms relative to information or messages on general and reproductive health care, including contraception and family planning methods toward demand satisfaction [53–55]. For instance, the regional groups in Myanmar show geographic inequalities in access to modern contraception or accessibility and quality of family planning services [53]. It is more likely that indicators such as physical distance, staying in hilly areas, and network access and/ or availability of media outlets might limit appropriate information or messages related to reproductive healthcare and services. This limitation could influence the demand satisfaction of the populace towards contraception and other family planning methods in the country [56]. Further, media information related concerns about the side effects, health consequences, and inconvenience of methods might negatively influence demand and satisfaction on contraceptive usage. The unpleasant thoughts of side effects, method-related and health concerns might be reasons that may account for low patronage or possibly discontinuation of use [57, 58].

Although extensive research has been carried out on family planning, little or no study emphasizes the satisfied demands of women who have been exposed to mass media. However, few studies [18, 19] have documented that women’s exposure to media is one of the two important factors that influence contraceptive use and promote health-related behaviour such as reproductive preferences. Other studies that hold a similar view with the current study are earlier works of Hailemariam [20] at an individual level and those of Abulmageed [21] at a national level, which found a relationship between mass media exposure and reduction in fertility levels. Ultimately, this narrative holds the view that the use of mass media positively increases the awareness about the need for fertility control [59].

This pioneering investigation of the effect of media exposure and family planning as an indicator of unmet needs for both Philippines and Myanmar, after adjusting for all the covariates, shows that media exposure has no significant effect on the unmet needs for both countries. However, this finding contradicts previous research [22, 23] that reported that exposure to newspaper/ magazine had a significant effect on unmet needs for family planning whereas those who had no information had a higher likelihood of their needs being unmet. The current finding is surprising because unmet needs for contraceptive and family planning methods is relatively higher in low-income developing countries like Philippines and Myanmar with low socio-economic indicators (e.g., poverty, low education) [60]. Therefore, in relatively low-income countries, the likelihood of few media establishments could restrict young people’s capacity because of low media education or information related to accessing contraceptives or seeking sexual and reproductive health services [61, 62]. Besides, the growing trend of premarital sexual relationship and unintended pregnancies noted in many South-East Asian countries (e.g., Indonesia, Myanmar, Nepal, Pakistan, Philippines) requires a greater need for contraceptives among the sexually active population [41, 63]. Methodological variations and the type of media exposure studied in previous studies might account for the inconsistencies. The theoretical implication is that mass media communication campaigns are highly capable of influencing family planning methods in both countries.

### Strength and limitations

This study is the first to investigate the effect of mass media exposure on family planning indicators in Philippines and Myanmar. It also provides insights into the effects of media exposure on demand satisfaction on family planning methods and on unmet needs for both countries that previous studies have ignored. The study is not without some limitations. First, the use of the large DHS dataset from cross-sectional perspective restricts causality from the noted outcomes. Second, because of the research design used, the possibility of social desirability associated with self-reporting and recall bias from the respondents cannot be ruled out. Also, the effects of the type of mass media exposure of the sample were not captured in the current study. Other limitations might include the alterations in the coding of the study variables, which could potentially generate data extraction errors. However, no substantial modifications were done during coding and extraction within the current study [47]. Despite these limitations, the sample size allows for generalizability of findings in the studied countries because data used were nationally representative.

## Conclusions

The study established a strong association between mass media exposure and the use of contraceptive and family planning as well as demand satisfaction among the sexually active population in Philippines and Myanmar. Using mass media exposure (e.g., local radio, television- electronic; newspapers) to increase both access and usage of contraceptives as well as other family planning methods in these countries could be pivotal towards the attainment of United Nations Sustainable Development Goal 3 (MDG 3) of improving maternal health. Therefore, the dissemination of contraceptive use and other family planning information through media channels could help minimize neonatal and infant deaths, unsafe abortions, and maternal deaths often noticed in low-income countries like Philippines and Myanmar. Continuous education or advocacy on reproductive health matters (i.e., on contraception/ family planning) using the mass media could serve as a public health strategy for health-promoting behaviours. Public-private-sector partnership for the establishment of media houses in these countries is encouraged. Future studies could target the influence of the type of media exposure on contraception and/ or family planning methods possibly through longitudinal designs to establish clear patterns or trends for effective reproductive health interventions and policy direction.

## Data Availability

The dataset supporting the conclusions of this article is available online at https://dhsprogram.com/data/

## Abbreviations

NDHS: National Demographic and Health Survey
MDHS: Demographic and Health Survey
aOR: Adjusted odds ratio
CI: Confidence interval
SDG: Sustainable Development Goal
PSA: Philippine Statistics Authority
